# Ethnic differences in use values and use patterns of *Parkia biglobosa *in Northern Benin

**DOI:** 10.1186/1746-4269-7-42

**Published:** 2011-12-07

**Authors:** Kourouma Koura, Jean C Ganglo, Achille E Assogbadjo, Clément Agbangla

**Affiliations:** 1 Faculty of Agronomic Sciences, University of Abomey-Calavi, Cotonou (01 PO Box 526), Benin; 2 Faculty of Sciences and Techniques, University of Abomey-Calavi, Cotonou (01 PO Box 526), Benin

**Keywords:** *Parkia biglobosa*, quantitative ethnobotany, use value, use pattern, Benin

## Abstract

**Background:**

African locust bean tree (*Parkia biglobosa*) is a multipurpose species used widely in arid Africa by local communities. The present study focused on ethnic differences in use values and use patterns of *P. biglobosa *in Northern Benin, where the species widely grows. The use values according to the various ethnic groups in the study area have been evaluated in detail for *P. biglobosa*.

**Methods:**

From 13 ethnic groups, 1587 people were interviewed in the study area using semi-structured questionnaires. Principal Component Analysis was applied to analyze the use value and the use patterns of *P. biglobosa *for the different ethnic groups.

**Results:**

All interviewees in the study area knew at least one use of *P. biglobosa*. The various uses identified were medicinal (47%), handicraft and domestic (3%), medico-magic (1%), veterinary (1%), cultural (1%), food (25%) and commercial (22%). The various parts involved in these types of uses were: fruits [shell (2%), pulp (22%) and seeds (36%)], bark (17%), leaves (9%), roots (3%), flowers (1%) and branches (10%). The ethnic group consensus values for *P. biglobosa *parts showed that the seeds are used the most. The interviewees diversity value (ID) and equitability value (IE) indicated that knowledge concerning *P. biglobosa *use was distributed homogeneously among the ethnic groups.

**Conclusions:**

*P. biglobosa *is well-known and used in different ways by the local populations in the study area. Local knowledge on the species is diversified and influenced by ethnic group. Ethnic differences in use values and use patterns of the species were evident in this study.

## Background

In the Sahelian and Sudanian zones of West Africa, woody perennial parklands are very important for food security, especially during food shortage and drought periods [1]. Within these parkland systems, *Parkia biglobosa *species has important socio-economic and cultural values for local people. Moreover, it is a food species whose importance is well recognized both regionally and internationally [2]. In Benin, *P. biglobosa *is an important tree species which generates non-timber forest products [3,4]. It is a basic and therapeutic food and is a source of wealth [5]. The pulp of the fruit pods is rich in sucrose and the seeds are rich in carbohydrates, proteins and lipids, thus constituting an important source of energy [6]. *P. biglobosa *is rated fifth important among thirty-one woody medicinal plants used in traditional medicine in Benin [4]. It is rated fourth from a list of eighteen priority food woody plants to preserve [7]. In association with crops, the species help to enrich physico-chemical soil characteristics which in turn help to increase crop yields.

It has been noted in Benin, that this species population is ageing. In addition, the natural regeneration of the species is very low [8,9]. Studies have indeed shown a decrease in the distribution of the species in Benin [10]. Due to the socio-economic and cultural importance of this species, communities tend to over utilize the plant species without taking into account the regeneration potential of the species [4,11].

A number of studies have been conducted on *P. biglobosa *in the West African sub-region and particularly in Benin. In West Africa, previous research has focused on the distribution of the species' populations, phenology, reproductive system, vegetative multiplication, biology and ecology of reproduction [2]. In the parklands in Benin, *P. biglobosa *was morphologically and structurally characterized and fruit production was assessed on the basis of floristic inventory and measurement of reproductive organ sizes [9]. From microbiological and physico-chemical points of view, it was noted that *P. biglobosa*' seeds could be fermented for the production of "*afitin*", "*iru*" and "*sonru*" (local names for condiments from the seeds of *P. biglobosa*) [12,13]. Volatile and aromatic compounds were also identified in these condiments in order to obtain, products of aromatic characteristics demanded by consumers. The place and role of this tree in the daily life of rural communities has been investigated [2,10,14,15].

Several research studies have been carried out on "use values" and "use patterns" of different species (such as *Adansonia digitata*, *Khaya senegalensis*, *Milicia excelsa*, *Caesalpinia bonduc*, *Sclerocarya birrea*) elsewhere [16-21] and in Benin [22-29]. However, little scientific information is available on quantitative descriptors of *P. biglobosa*'s utilization, especially the "use values" and "use patterns" of the species by local communities of Northern Benin. The present study was set up to address the following questions: (1) What ethnobotanical knowledge do local communities have on the use of *P. biglobosa*? (2) What factors (ethnic group, sex and age) can be used to predict local communities' knowledge on the use of *P. biglobosa*? These questions are based on quantitative measures currently used in ethnobotanical studies [18,30].

## Methods

### Study area

**Figure 1:**
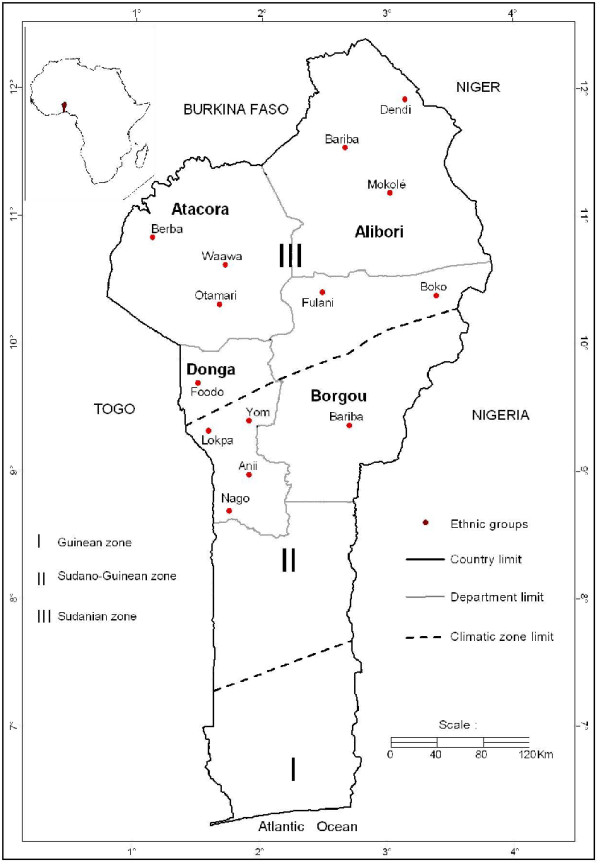
**Study area and ethnic groups studied in Northern Benin**. This map has been devised and carried out by the authors.

The study was carried out in Northern Benin, where the species widely grows. Northern Benin has four Departments (administrative subdivisions): Alibori, Donga, Borgou and Atacora (Figure 1). Each Department is divided into municipalities and each municipality has several villages. These Departments occupy a total area of 83723 km^2^, or 74% of the total area of Benin (112622 km^2^). They belong to two climatic zones of Benin: the Sudanian climatic zone (between 9°45' - 12°25' N) and the Sudano-Guinean climatic zone (between 7°30' - 9°45' N). The rainfall in these two zones is unimodal. The Sudanian climatic zone is a woodland and savanna region with ferruginous soils. The mean annual temperature is 35°C. The mean annual rainfall varies between 900 mm - 1100 mm. The Sudano-Guinean climatic zone is a transitional zone between the sub-humid Guinean zone and the Sudanian zone. This zone is characterized by a vegetation mosaic of forest islands, gallery forests and savannas. The population of the study area was estimated at 2,144,743 inhabitants and the farm population was 1,637,434 inhabitants [31]. The dominant ethnic groups in the study area are: Bariba, Fulani, Dendi, Mokolé, Berba, Waama, Lokpa, Yom and Nago. Other groups such as Otamari, Boko, Anii and Foodo are also present. Livelihood activities carried out by the people of these ethnic groups include agriculture, ranching, fishing, hunting, processing of agricultural products, trade and craft. The processing of agricultural products is mainly practiced by women individually or in groups with handicraft equipment. The main processed materials are *Vitellaria paradoxa *nuts (transformed into butter), seeds of *Parkia biglobosa *(transformed into a food condiment with a ready market), the seeds of *Sorghum bicolor *(transformed into an alcoholic beverage and also used in some traditional ceremonies), pods of *Arachis hypogea *(transformed into oil).

### Data collection

All municipalities of the study area and all villages of the selected municipalities were subjected into a hierarchical cluster analysis based on demographic data collected at the National Institute of Statistics and Economic Analyses of the Republic of Benin.

In each selected village, the sample size (n) used for the study was computed based on the following formula [32]:

$n=\frac{{⋃}_{1-\alpha \;/\;2}^{2}p\left(1-p\right)}{d^{2}}$ where,

n is the total number of surveyed people in the study;

${⋃}_{1-\alpha \;/\;2}^{2}$is the value of the normal random variable for a probability value of *α *= 0.05, ${⋃}_{1-\alpha \;/\;2}^{}=1.96$;

p is the estimated proportion of people in the village which was involved in the production, processing and/or marketing of *P. biglobosa*. During an exploration phase, it was noticed that most of the rural households used the *P. biglobosa *species as producers or processors or traders. In this study, p was taken as the ratio of agricultural population and total population of each selected village;

d is the expected error margin of any parameter to be computed from the survey, which is fixed at 0.1.

After estimating the sample size, local people were randomly selected. The target groups consisted of traders, traditional healers and farmers. Table 1 summarizes the sample size of people surveyed by Department, ethnic group, sex and age group. Semi-structured interviews surveys were conducted among various people from 13 ethnic groups in the study area, namely Berba, Waama, Otamari, Anii, Nago, Lokpa, Yom, Foodo, Dendi, Mokolé, Bariba, Boko and Fulani. The questionnaire was tailored for each category of target group. The questionnaire evaluated information concerning demographic characteristics (name, sex, age, ethnic group and main activity) and use forms of *P. biglobosa*.

**Table 1:** Sample size per Department, ethnic group, sex and age.

Department	Sample size	Ethnic group	Sex		Age		Total per ethnic group
				
			Men	Women	≥ 40 years	< 40 years	
Atacora	401	Berba	36	34	59	11	70
		Waama	152	89	182	59	241
		Otamari	62	28	59	31	90
Donga	585	Anii	64	2	55	11	66
		Nago	57	6	53	10	63
		Lokpa	257	29	173	113	286
		Yom	99	30	97	32	129
		Foodo	40	1	29	12	41
Alibori	311	Dendi	59	29	76	12	88
		Mokolé	43	15	46	12	58
		Bariba	122	43	107	58	165
Borgou	290	Boko	42	11	43	10	53
		Fulani	93	21	98	16	114
		Bariba	89	34	101	22	123
Total	1587	-	1215	372	1178	409	1587

### Data analysis

For ethnobotanical data analysis, the following indices were used: (1) the interviewee diversity value (ID); (2) the interviewee equitability value (IE); (3) the consensus value for plant parts (CPP); (4) the use diversity value (UD); (5) the use equitability value (UE) and (6) the consensus value for the form of use (CMU). Table 2 provides a detailed explanation of each index. These parameters indicate how the species is used and how the knowledge of these uses is distributed among the interviewees (table 2). To compute the use diversity value (UD), specific uses of *P. biglobosa *were classified into different categories of uses.

**Table 2:** Indices of knowledge and uses calculated for *P. biglobosa*.

Index	Calculation	Description	Reference
Interviewee diversity value (ID) ID = Ux/Ut	ID, number of uses-citations by a given interviewee (Ux) divided by the total number of uses (Ut)	Measures how many interviewees used *Parkia biglobosa *and how its uses are distributed among the interviewees	[17]
Interviewee equitability value (IE) IE = ID/IDmax	IE, interviewee diversity value (ID) divided by this index's maximum value (IDmax)	Measures the degree of homogeneity of the interviewee's knowledge	[17]
Consensus value for plant parts (CPP) CPP = Px/Pt	CPP, number of times a given plant part was cited (Px) divided by the total number of citations of all parts (Pt)	Measures the degree of agreement among interviewees concerning the plant part used	[18]
Use diversity value (UD) UD = Ucx/Uct	UD, number of indications recorded by category (Ucx) divided by the total number of indications for all categories (Uct)	Measures the importance of the use categories and how they contribute to the total value of uses	[17]
Use equitability value (UE) UE = UD/UDmax	UE, use-diversity value (UD) divided by the index's maximum value (UDmax)	Measures the degree of homogeneity of knowledge about use categories	[17]
Consensus value for the form of use (CMU) CMU = Mx/Mt	CMU, number of citations for a given form of use (Mx) divided by the total number of citations for all forms (Mt)	Measures the degree of agreement among interviewees concerning the form of use of *Parkia biglobosa*	[18]

The normality and homogeneity of the ID and IE indices calculated were audited and the non parametric Kruskal-Wallis test, using Minitab, was carried out to assess significant differences related to sex, age and ethnic group. The consensus value for plant parts (CPP) and the consensus value for the form of usage (CMU) calculated per ethnic group were subjected to Principal Component Analysis (PCA) using SAS 9.1 software to better describe the relationship between these use values and the ethnic groups.

Moreover, the retention degree of the medicinal recipes in each ethnic group was determined from the diversity index of Simpson (1/D); which is calculated from the Simpson index (D) as follows:

$$D=\overset{S}{\underset{i=1}{\sum }}\frac{ni\left(ni-1\right)}{N\left(N-1\right)}$$

with S being the total number of interviewees per ethnic group; N, arithmetic sum of ni recipes and ni, the number of recipes per interviewee. The diversity index of Simpson (1/D) ranges from 1 to S. If 1/D tends to S, the recipes are shared by everyone in the group. When 1/D approached 1, the recipes were only retained by a small group of people.

## Results

### Diversity and distribution of knowledge among interviewees

All farmers and traditional healers surveyed in the study area used *P. biglobosa*. The values for total diversity value (ID) and total equitability value (IE) were generally high (> 0.50) and indicated that knowledge related to *P. biglobosa *was distributed homogeneously within the target groups (table 3). The additional file 1; 2; 3 and 4 give more detail on the quantitative measurements of knowledge about *P. biglobosa *in the different Department of the study area. Ethnic group, sex, age and locality (Department) were important factors relating to knowledge distribution. In the Borgou Department, the values of these indices were significantly different among ethnic groups (p = 0.001). These differences, within Borgou, were due to the fact that some interviewees had little knowledge on the species. This was the case in young Fulani women. In the Atacora Department, the ID and IE values for traditional healers differed significantly. These differences were due to the fact that knowledge of *P. biglobosa *were more diversified and more distributed within Waama aged men (ID = 0.10 and IE = 0.40) than within young Otamari men (ID = 0.008 and IE = 0.03). In the Donga Department, the ID and IE values calculated for the farmers were significantly different among ethnic groups. These differences were due to the fact that knowledge of *P. biglobosa *was more diversified and more distributed within Lokpa men (ID = 0.36 and IE = 0.52) than within young Nago and Foodo men (ID = 0.04 and IE = 0.06 for each ethnic group). In the study area, processing activities and sale of organs and products from *P. biglobosa *were mainly conducted by women.

**Table 3:** Summary of quantitative measurements of knowledge about *P. biglobosa*

Department	Farmers				Traditional Healers			
	
	Total number of interviewees	Number of uses cited	Total ID (p)	Total IE (p)	Total number of interviewees	Number of uses cited	Total ID (p)	Total IE (p)
Alibori	219	25	0.99 (0.308)	0.99 (0.308)	27	19	0.98 (0.139)	0.98 (0.139)
Borgou	218	47	0.52 (0.001)	0.81 (0.001)	20	40	0.71 (< 0.001)	0.83 (< 0.001)
Atacora	230	19	0.58 (0.151)	0.79 (0.151)	42	42	0.23 (0.020)	0.88 (0.020)
Donga	488	76	0.61 (0.012)	0.87 (0.012)	41	47	0.43 (0.362)	0.51 (0.362)

* ID = Interviewee Diversity value; IE = Interviewee Equitability value; p = level of signification

### Use diversity value

Uses of *P. biglobosa *were grouped into different categories: medicinal (47% of the interviewees), food (25%), commercial (22%), handicrafts and domestic (3%), medico-magic (1%), veterinary (1%) and cultural (1%). In each Department, whatever the ethnic group, most interviewees mentioned medicinal uses (table 4).

**Table 4:** Use diversity value (UD) and equitability value (UE) according to various uses of *P. biglobosa*

Category of use	Alibori						Borgou						Atacora						Donga									
	
	Dendi		Mokolé		Bariba		Bariba		Fulani		Boko		Berba		Waama		Otamari		Anii		Nago		Lokpa		Yom		Foodo	
	
	UD	UE	UD	UE	UD	UE	UD	UE	UD	UE	UD	UE	UD	UE	UD	UE	UD	UE	UD	UE	UD	UE	UD	UE	UD	UE	UD	UE
Medicinal	0.72	1	0.75	1	0.72	1	0.62	1	0.69	1	0.63	1	0.76	1	0.54	1	0.46	1	0.67	1	0.6	1	0.64	1	0.70	1	0.59	1
Medico-magic	-	-	-	-	-	-	0.12	0.19	0.14	0.2	0.05	0.08	-	-	0.14	0.26	-	-	0.12	0.17	0.11	0.19	0.10	0.16	0.14	0.19	0.09	0.15
Veterinary	-	-	-	-	-	-	-	-	-	-	-	-	-	-	-	-	0.09	0.2	-	-	0.02	0.04	0.03	0.05	-	-	-	-
Cultural	-	-	-	-	-	-	0.02	0.03	-	-	0.03	0.04	0.04	0.05	0.06	0.11	-	-	-	-	-	-	0.03	0.05	-	-	-	-
Handicrafts and domestic	0.04	0.06	0.04	0.06	0.04	0.06	0.05	0.08	0.05	0.08	0.05	0.08	0.04	0.05	0.09	0.16	0.09	0.2	0.07	0.10	0.11	0.19	0.09	0.13	0.03	0.04	0.14	0.23
Food	0.12	0.17	0.13	0.17	0.12	0.17	0.09	0.14	0.02	0.03	0.11	0.17	0.08	0.11	0.09	0.16	0.18	0.4	0.07	0.10	0.04	0.07	0.05	0.08	0.05	0.08	0.09	0.15
Commercial	0.12	0.17	0.08	0.11	0.12	0.17	0.10	0.17	0.10	0.15	0.13	0.21	0.08	0.11	0.09	0.4	0.18	0.4	0.07	0.10	0.11	0.19	0.05	0.08	0.08	0.12	0.09	0.15

The medicinal uses of the species were the most diversified. Particularly, the digestive system diseases (diarrhoea, dysentery, abdominal pain), diseases of the cardiovascular system, injuries and burns, infectious diseases (shingles, malaria, abscesses, yellow fever, scabies, measles, chicken-pox, edema, jaundice), pediatric pathologies, symptoms and syndromes (malaise, tiredness, headaches, hip pain, ache, rheumatism, excessive weight loss, elephantiasis, onset of paralysis) were reported by most interviewees.

The food uses (transformation of the seeds in condiment, consumption of pulp) and commercial uses (sale of seeds, condiment from seeds, pulp, sponge from roots, wood fire) were observed for all ethnic groups surveyed. These findings demonstrate the importance of *P. biglobosa *in eating habits as well as in the improvement of agricultural households' income. The seeds and pulp (the most marketed plant parts) are an important source of income for farmers. They sell the seeds to the women who invest in the processing of seeds in food condiment. This condiment is appreciated in the study area.

### *Parkia biglobosa *plant parts used

**Figure 2:**
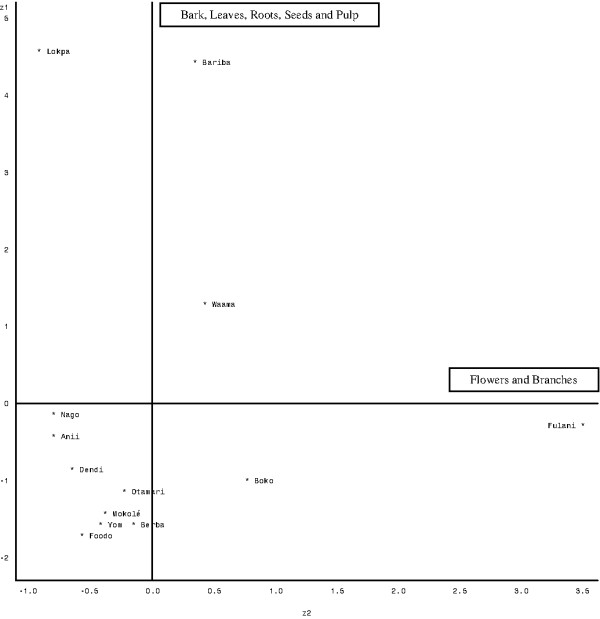
**Projection of targeted ethnic groups in the system axis defined by the different organs**.

The *P. biglobosa *parts used were: fruits [shell (2%), pulp (22%) and seeds (36%)], bark (17%), leaves (9%), roots (3%), flowers (1%) and branches (10%). The fruits (seeds and pulp), bark, leaves and branches were the organs most used. The results of the Principal Component Analysis (PCA) performed on the overall consensus values on *P. biglobosa *parts regarding ethnic groups (table 5) showed that the first two axes accounted for 75.22% of the total variation. Therefore, only these axes were retained to describe the relationship between *P. biglobosa *parts and ethnic groups. Table 6 shows the correlation coefficient between the different parts and the two axes. The first axis shows a positive link between bark, leaves, roots, seeds and pulp. Axis 2 shows the positive link between flowers and branches. Moreover, Figure 2 shows the projection of different ethnic groups into the system axes 1 and 2. From these observations, it can be deduced that Lokpa, Waama and Bariba ethnic groups assigned a high consensus value for bark, leaves, roots, seeds and pulp. In contrast, Nago, Anii, Dendi, Otamari, Mokolé, Foodo, Yom, Berba, Boko and Fulani assigned low value to bark and leaves. Ethnobotanical consensus values for branches were high for Fulani, Waama and Boko but low for other ethnic groups.

**Table 5:** Consensus values for *Parkia biglobosa *parts (CPP)

Parts	Alibori			Borgou			Atacora			Donga				
	
	Dendi	Mokolé	Bariba	Bariba	Fulani	Boko	Berba	Waama	Otamari	Anii	Nago	Lokpa	Yom	Foodo
Bark	0.25	0.235	0.271	0.114	0.068	0.063	0.198	0.146	0.118	0.173	0.183	0.201	0.189	0.248
Leaves	0.156	0.139	0.133	0.089	0.041	0.043	0.102	0.063	0.055	0.07	0.107	0.088	0.059	0.203
Roots	0.04	0.036	0.069	0.018	0.007	0.013	-	0.023	0.059	0.096	0.127	0.023	0.016	0.03
Seeds	0.375	0.398	0.352	0.253	0.341	0.275	0.353	0.402	0.349	0.368	0.317	0.414	0.519	0.391
Pulp	0.156	0.054	0.09	0.253	0.341	0.275	0.198	0.193	0.261	0.272	0.183	0.255	0.205	0.12
Shells of fruits	0.022	0.018	0.024	0.06	0.032	0.02	-	-	0.004	0.018	0.048	0.01	0.011	0.008
Flowers	-	-	-	-	0.007	-	-	-	-	-	-	-	-	-
Branches	-	0.12	0.06	0.213	0.161	0.311	0.150	0.173	0.155	0.004	0.036	0.009	-	-

**Table 6:** Correlation between *P. biglobosa *parts and PCA axes

Parts	Axis 1	Axis 2
Bark	0.954	-0.195
Leaves	0.952	-0.158
Roots	0.733	-0.306
Seeds	0.915	0.006
Pulp	0.876	0.219
Shells	0.714	0.230
Flowers	-0.041	0.897
Branches	0.426	0.551

### Forms of use of *Parkia biglobosa*

**Figure 3:**
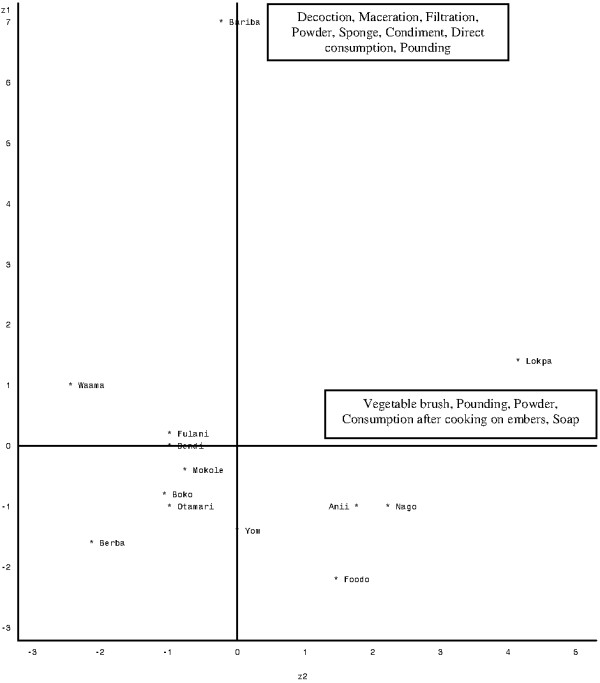
**Projection of targeted ethnic groups in the system axis defined by the forms of use**.

The different forms of use of *P. biglobosa *in the study area were: decoction, maceration, filtration, powder, boiled pulp, sponge, vegetable brush (toothpick), condiment derivative of seeds/potash, direct consumption, pounding, small cushion (leaves of *P. biglobosa *rolled as a cushion), chewing, consumption after cooking on embers, soap and frankincense (table 7). The results of the Principal Component Analysis (PCA) performed on the overall consensus values per form of use by ethnic groups, showed that the first two axes explained 60.31% of the total variation. Therefore, only these axes were used to describe the relationship between use forms and ethnic groups. Figure 3 shows the projection of different ethnic groups into the system axes 1 and 2. The correlation coefficient between the different use forms and the first axis showed a positive link between decoction, maceration, filtration, condiment, powder, sponge, pounding and direct consumption. Axis 2 shows a positive link between vegetable brush (toothpick), pounding, powder, consumption after cooking on embers and soap. We therefore deduce that ethnobotanical consensus values for decoction, maceration, filtration, condiment, powder, sponge, pounding and direct consumption use forms were high for Bariba, Dendi, Fulani, Waama and Lokpa but low for other ethnic groups. Vegetable brush (toothpick), pounding, powder, consumption after cooking on embers, soap consensus values were high for Lokpa, Anii, Foodo and Yom but low for other ethnic groups.

**Table 7:** Consensus values for *Parkia biglobosa *forms of usage (CMU)

Forms of use	Alibori			Borgou			Atacora			Donga				
	
	Dendi	Mokolé	Bariba	Bariba	Fulani	Boko	Berba	Waama	Otamari	Anii	Nago	Lokpa	Yom	Foodo
Decoction	0.274	0.289	0.293	0.195	0.141	0.257	0.245	0.367	0.151	0.103	0.103	0.099	0.16	0.123
Maceration	0.137	0.104	0.133	0.047	0.025	0.029	0.06	0.036	0.086	0.06	0.103	0.072	0.08	0.046
Filtration	0.16	0.121	0.102	0.053	0.075	0.214	0.007	0.036	-	-	0.019	0.041	-	-
Powder	0.032	0.064	0.038	0.005	0.05	-	0.03	0.007	0.046	0.128	0.058	0.051	0.024	0.077
Boiled pulp	0.137	0.116	0.091	0.045	0.033	0.029	0.323	0.281	0.355	0.12	0.116	0.206	0.096	0.169
Sponge	0.005	0.006	0.087	-	-	-	-	-	-	0.085	0.129	0.01	0.008	0.015
Vegetable brush (toothpick)	0.005	0.006	0.022	-	-	-	0.012	0.007	0.007	0.154	0.2	0.362	0.424	0.308
Seeds/potash derived condiment	0.251	0.295	0.233	0.321	0.394	0.243	0.307	0.245	0.349	0.205	0.148	0.041	0.08	0.031
Direct consumption	-	-	-	0.216	0.266	0.186	-	-	-	-	-	-	-	-
Pounding	-	-	-	0.118	0.017	0.043	-	-	-	0.12	0.097	0.111	0.128	0.169
Small cushion	-	-	-	-	-	-	0.002	0.014	-	-	-	-	-	-
Chewing	-	-	-	-	-	-	0.014	0.007	0.007	-	-	-	-	-
Consumption after cooking on embers	-	-	-	-	-	-	-	-	-	0.017	0.006	0.004	-	0.015
Soap	-	-	-	-	-	-	-	-	-	0.009	0.013	0.002	-	0.046
Frankincense	-	-	-	-	-	-	-	-	-	-	0.006	-	-	-

The Simpson's diversity indices for different ethnic groups were significantly lower than S, i.e. the number of interviewees per ethnic group (table 8), meaning that each respondent knew practically at least one use of *P. biglobosa*. Each respondent had their way of using the species. Taking into account the Simpson's diversity indices and coefficients of variation, it can be concluded that the recipes are diversified and vary according to ethnic group.

**Table 8:** Simpson's diversity indexes calculated by ethnic group in the study area

Parameters calculated	Alibori			Borgou			Atacora			Donga				
	
	Dendi	Mokolé	Bariba	Bariba	Fulani	Boko	Berba	Waama	Otamari	Anii	Nago	Lokpa	Yom	Foodo
Number of interviewees by ethnic group (S)	69	54	123	50	39	58	47	67	266	64	46	260	109	40
Simpson's diversity index (1/D)	5.66	11.04	6.70	2.72	1.54	4.12	2.76	6.12	5.88	11.22	1.41	12.57	11.88	8.21

## Discussion

This work has demonstrated that *Parkia biglobosa *is a multipurpose species in Benin. The species is used in multiple ways. The ethnobotanical indices (quantitative measures) allowed for the reaching of consensus among interviewees on the uses of the species. The approach assumes that there is no relationship between what the interviewee says and what he or she actually does. These quantitative measures reflect a consensus among interviewees, based on the understanding that culture represents shared knowledge [16]. The importance and usefulness of collecting local communities' knowledge lie in the fact that such information could help determining the true value of the species, leading to more rational decisions about its sustainable utilization. Knowledge of the local usage of vegetable resources is essential for the elaboration of conservation strategies [33]. This is the first time that the use values according to various ethnic groups in the study area have been evaluated in detail for *P. biglobosa*.

Overall, we found significant ethnic variation in knowledge and use values of *P. biglobosa*, as has been found for *Tamarindus indica *[25], *Adansonia digitata *[22,27,34] and *Caesalpinia bonduc *[26]. Local knowledge varied not only according to different ethnic groups but also according to the age and sex of individuals interviewed. The results confirm the significant age and gender differences in the use of medicinal plants shown by [19-21] and [35].

This work also identified the various diseases treated by *P. biglobosa *and the recipes used by different ethnic groups, which falls under ethno-herbal medicine. All organs of the tree were widely recognized as being used for food, medicinal, medico-magical, cultural and others. All parts of the tree (roots to leaves) are valued and used differently by people. Fermented seeds and yellow floury pulp (rich in saccharose) are both highly appreciated as food. In a large part of West Africa [2] particularly in Togo [36], Nigeria [37], Mali [38], Benin [5,15], the food condiment from *P. biglobosa *seeds is the main seasoning sauce. The medicinal' uses of the species is the most diversified. This is general in West Africa where all organs of the species are used as main recipe or in combination with other plants in the treatment of several diseases [39]. Biochemical analysis has shown active compounds in different organs which explain and confirm several indigenous medicinal uses. The organs of *P. biglobosa *are rich in various components such as flavonoid aglycones, fatty acids, tannins, reducing compounds (fructose and glucose), carbohydrates and anthocyanosides [2]. The *P. biglobosa *products are also rich in protein [2,40] and can therefore be used as protein supplements in diets based mainly on cereals. Therefore, *P. biglobosa *has medicinal potential on which research and development could be derived to develop standardized phytomedicines and reduce the pressure on the species in its natural habitats. It is therefore important to value the food condiment from seeds of *P. biglobosa *since it has a certain nutritional value unlike flavoring imported mainly consisting of glutamate.

The important functions of *P. biglobosa *especially in the food and traditional medicine are the main reasons justifying its conservation *in situ*. However, *P. biglobosa *is a vulnerable tree species since all plant parts are collected for various purposes. The fruits (seeds and pulp), bark, leaves and branches are the organs most used. Harvesting of bark has more negative impacts on habitat and population of the species than harvesting leaves and fruits [41-45]. However, harvesting of leaves and fruits may also have a negative impact on the regeneration process because the maintenance of regeneration capacity also depends on the maintenance of key regeneration principles such as pollination, development and dispersal of seeds, germination and plant growth. It is therefore necessary to consider strategies for the conservation of valuable species such as *P. biglobosa*.

## Conclusion

From the present study, we can conclude that *Parkia biglobosa *is a well-known resource and used in different ways by locals in Benin. Local knowledge on the species is diversified and dependent on sex, age and ethnic group. Knowledge is homogeneously distributed within ethnic groups. All parts of *P. biglobosa *are used for various purposes. Ethnic differences in use values and use patterns of the species were clearly observed in this study. Among all the studied ethnic groups, Lokpa, Waama and Bariba had the highest level of knowledge on *P. biglobosa *parts and forms of use while the other ethnic groups had limited level of knowledge. Local knowledge varied not only according to ethnic group but also according to the age and sex of individuals. For instance, knowledge of *P. biglobosa *were more diversified and more distributed within older Waama men and Lokpa men than within young Otamari men, Fulani women and young Nago and Foodo men.

## Consent statement

I attest to the fact that all authors listed on the title page have read the manuscript, attest to the validity and legitimacy of the data and its interpretation, and agree to its submission to *Journal of Ethnobiology and Ethnomedicine*. Written informed consent was obtained from the participants of this study.

## Competing interests

The authors declare that they have no competing interests.

## Authors' contributions

KK designed and performed the field work, analyzed and drafted the manuscript. AEA gave conceptual advice, read and improved the drafted manuscript. JCG and CA supervised the work and improved the manuscript. All authors have read and approved the final manuscript.

## Supplementary Material

Additional file 1 **Quantitative measurements of knowledge about *P. biglobosa *in Alibori Department**. Click here for file

Additional file 2 **Quantitative measurements of knowledge about *P. biglobosa *in Borgou Department**. Click here for file

Additional file 3 **Quantitative measurements of knowledge about *P. biglobosa *in Atacora Department**. Click here for file

Additional file 4 **Quantitative measurements of knowledge about *P. biglobosa *in Donga Department**. The additional file 1; 2; 3 and 4 give more detail on how the data were analyzed by ethnic group, gender group and age group. Click here for file
