# Supplementary material for: Ethnic differences in use values and use patterns of Parkia biglobosa in Northern Benin
Source: J Ethnobiol Ethnomed. 2011 Dec 7;7:42. doi: 10.1186/1746-4269-7-42 (PMC3251525; doi:10.1186/1746-4269-7-42)
Supplement: Additional file 1 — Quantitative measurements of knowledge about P. biglobosa in Alibori Department. [file 1746-4269-7-42-S1.PDF]

Additional file 1 Quantitative measurements of knowledge about *P. biglobosa* in Alibori Department

	Farmers	Traditional Healers
Total number of interviewees	219	27
Number of uses cited	25	19
Interviewee diversity value (ID)	Mean (Standard deviation)	Mean (Standard deviation)
Total ID	0.99 (0.02) a	0.98 (0.03) a
Total ID for men	0.99 (0.02) a	0.98 (0.03) a
ID Men Dendi	0.64 (0.56) a	0.32 (0.55) a
ID Men Dendi ≥ 40 years old	0.63 (0.55) a	0.32 (0.55) a
ID Men Dendi < 40 years old	0.53 (0.46) a	0.00 (0.00) a
ID Men Bariba	0.64 (0.56) a	0.33 (0.58) a
ID Men Bariba ≥ 40 years old	0.60 (0.52) a	0.33 (0.58) a
ID Men Bariba < 40 years old	0.61 (0.54) a	0.00 (0.00) a
ID Men Mokolé	0.63 (0.54) a	0.33 (0.58) a
ID Men Mokolé ≥ 40 years old	0.61 (0.53) a	0.33 (0.58) a
ID Men Mokolé < 40 years old	0.52 (0.46) a	0.00 (0.00) a
Interviewee equitability value (IE)	Mean (Standard deviation)	Mean (Standard deviation)
Total IE	0.99 (0.02) a	0.98 (0.03) a
Total IE pour Hommes	0.99 (0.02) a	0.98 (0.03) a
IE Men Dendi	0.64 (0.56) a	0.32 (0.55) a
IE Men Dendi ≥ 40 years old	0.63 (0.55) a	0.32 (0.55) a
IE Men Dendi < 40 years old	0.53 (0.46) a	0.00 (0.00) a
IE Men Bariba	0.64 (0.56) a	0.33 (0.58) a
IE Men Bariba ≥ 40 years old	0.60 (0.52) a	0.33 (0.58) a
IE Men Bariba < 40 years old	0.61 (0.54) a	0.00 (0.00) a
IE Men Mokolé	0.63 (0.54) a	0.33 (0.58) a
IE Men Mokolé ≥ 40 years old	0.61 (0.53) a	0.33 (0.58) a
IE Men Mokolé < 40 years old	0.52 (0.46) a	0.00 (0.00) a

In a single column, for each index, the values followed by the same letter are not significantly different (Kruskal-Wallis test)
