# Supplementary material for: Ethnic differences in use values and use patterns of Parkia biglobosa in Northern Benin
Source: J Ethnobiol Ethnomed. 2011 Dec 7;7:42. doi: 10.1186/1746-4269-7-42 (PMC3251525; doi:10.1186/1746-4269-7-42)
Supplement: Additional file 2 — Quantitative measurements of knowledge about P. biglobosa in Borgou Department. [file 1746-4269-7-42-S2.PDF]

Additional file 2 Quantitative measurements of knowledge about *P. biglobosa* in Borgou Department

	Farmers	Traditional Healers
Total number of interviewees	218	20
Number of uses cited	47	40
Interviewee diversity value (ID)	Mean (Standard deviation)	Mean (Standard deviation)
Total ID	0.52 (0.12) a	0.71 (0.13) a
Total ID for Women	0.17 (0.06) cde	0.00 (0.00) c
ID Women Boko	0.04 (0.06) de	0.00 (0.00) c
ID Women Boko ≥ 40 years old	0.04 (0.06) de	0.00 (0.00) c
ID Women Boko < 40 years old	0.00 (0.00) e	0.00 (0.00) c
ID Women Bariba	0.14 (0.08) cde	0.00 (0.00) c
ID Women Bariba ≥ 40 years old	0.14 (0.08) cde	0.00 (0.00) c
ID Women Bariba < 40 years old	0.00 (0.00) e	0.00 (0.00) c
ID Women Fulani	0.05 (0.08) de	0.00 (0.00) c
ID Women Fulani ≥ 40 years old	0.05 (0.07) de	0.00 (0.00) c
ID Women Fulani < 40 years old	0.02 (0.03) e	0.00 (0.00) c
Total ID for Men	0.49 (0.13) a	0.71 (0.13) a
ID Men Boko	0.11 (0.19) de	0.17 (0.30) bc
ID Men Boko ≥ 40 years old	0.11 (0.19 de	0.17 (0.30) bc
ID Men Boko < 40 years old	0.00 (0.00) e	0.00 (0.00) c
ID Men Bariba	0.45 (0.06) ab	0.44 (0.10) ab
ID Men Bariba ≥ 40 years old	0.38 (0.03) abc	0.44 (0.10) ab
ID Men Bariba < 40 years old	0.08 (0.12) de	0.00 (0.00) c
ID Men Fulani	0.30 (0.13) abcd	0.46 (0.25) ab
ID Men Fulani ≥ 40 years old	0.26 (0.08) bcde	0.36 (0.26) abc
ID Men Fulani < 40 years old	0.19 (0.06) bcde	0.17 (0.25) bc
Interviewee equitability value (IE)	Mean (Standard deviation)	Mean (Standard deviation)
Total IE	0.81 (0.18) a	0.83 (0.15) a
Total IE for Women	0.27 (0.10) cde	0.00 (0.00) c
IE Women Boko	0.06 (0.10) de	0.00 (0.00) c
IE Women Boko ≥ 40 years old	0.06 (0.10) de	0.00 (0.00) c
IE Women Boko < 40 years old	0.00 (0.00) e	0.00 (0.00) c
IE Women Bariba	0.22 (0.12) de	0.00 (0.00) c
IE Women Bariba ≥ 40 years old	0.22 (0.12) de	0.00 (0.00) c
IE Women Bariba < 40 years old	0.00 (0.00) e	0.00 (0.00) c
IE Women Fulani	0.08 (0.12) de	0.00 (0.00) c
IE Women Fulani ≥ 40 years old	0.07 (0.11) de	0.00 (0.00) c
IE Women Fulani < 40 years old	0.04 (0.04) e	0.00 (0.00) c
Total IE for Men	0.76 (0.21) a	0.83 (0.15) a
IE Men Boko	0.17 (0.29) de	0.20 (0.35) bc
IE Men Boko ≥ 40 years old	0.17 (0.29) de	0.20 (0.35) bc
IE Men Boko < 40 years old	0.00 (0.00) e	0.00 (0.00) c
IE Men Bariba	0.63 (0.09) ab	0.52 (0.11) ab
IE Men Bariba ≥ 40 years old	0.58 (0.05) abc	0.52 (0.11) ab
IE Men Bariba < 40 years old	0.12 (0.19) de	0.00 (0.00) c
IE Men Fulani	0.47 (0.19) abcd	0.55 (0.30) ab
IE Men Fulani ≥ 40 years old	0.40 (0.13) bcde	0.43 (0.31) ab
IE Men Fulani < 40 years old	0.19 (0.18) de	0.20 (0.29) bc

In a single column, for each index, the values followed by the same letter are not significantly different (Kruskal-Wallis test)
