# Supplementary material for: Ethnic differences in use values and use patterns of Parkia biglobosa in Northern Benin
Source: J Ethnobiol Ethnomed. 2011 Dec 7;7:42. doi: 10.1186/1746-4269-7-42 (PMC3251525; doi:10.1186/1746-4269-7-42)
Supplement: Additional file 3 — Quantitative measurements of knowledge about P. biglobosa in Atacora Department. [file 1746-4269-7-42-S3.PDF]

**Additional file 3** Quantitative measurements of knowledge about *P. biglobosa* in Atacora Department

| | Farmers | Traditional Healers |
|---|---|---|
| Total number of interviewees | 230 | 42 |
| Number of uses cited | 19 | 42 |
| Interviewee diversity value (ID) | Mean (Standard deviation) | Mean (Standard deviation) |
| Total ID | 0.58 (0,16) a | 0.23 (0.04) a |
| Total ID for Women | 0.25 (0.03) a | 0.00 (0.00) b |
| ID Women Berba | 0.10 (0.18) a | 0.00 (0.00) b |
| ID Women Berba ≥ 40 years old | 0.07 (0.12) a | 0.00 (0.00) b |
| ID Women Berba < 40 years old | 0.10 (0.18) a | 0.00 (0.00) b |
| ID Women Otamari | 0.07 (0.12) a | 0.00 (0.00) b |
| ID Women Otamari ≥ 40 years old | 0.05 (0.09) a | 0.00 (0.00) b |
| ID Women Otamari < 40 years old | 0.07 (0.12) a | 0.00 (0.00) b |
| ID Women Waama | 0.17 (0.15) a | 0.02 (0.04) b |
| ID Women Waama ≥ 40 years old | 0.17 (0.15) a | 0.00 (0.00) b |
| ID Women Waama < 40 years old | 0.17 (0.15) a | 0.02 (0.04) b |
| Total ID for Men | 0.58 (0.16) a | 0.22 (0.05) a |
| ID Men Berba | 0.14 (0.24) a | 0.08 (0.14) b |
| ID Men Berba ≥ 40 years old | 0.14 (0.24) a | 0.08 (0.14) b |
| ID Men Berba < 40 years old | 0.09 (0.15) a | 0.00 (0.00) b |
| ID Men Otamari | 0.07 (0.12) a | 0.03 (0.05) b |
| ID Men Otamari ≥ 40 years old | 0.07 (0.12) a | 0.03 (0.05) b |
| ID Men Otamari < 40 years old | 0.07 (0.12) a | 0.01 (0.01) b |
| ID Men Waama | 0.44 (0.39) a | 0.13 (0.11) ab |
| ID Men Waama ≥ 40 years old | 0.42 (0.38) a | 0.10 (0.09) ab |
| ID Men Waama < 40 years old | 0.23 (0.20) a | 0.04 (0.07) b |
| Interviewee equitability value (IE) | Mean (Standard deviation) | Mean (Standard deviation) |
| Total IE | 0.79 (0.21) a | 0.88 (0.14 a |
| Total IE for Women | 0.33 (0.04) a | 0.00 (0.00) b |
| IE Women Berba | 0.14 (0.25) a | 0.00 (0.00) b |
| IE Women Berba ≥ 40 years old | 0.09 (0.16) a | 0.00 (0.00) b |
| IE Women Berba < 40 years old | 0.14 (0.25) a | 0.00 (0.00) b |
| IE Women Otamari | 0.09 (0.16) a | 0.00 (0.00) b |
| IE Women Otamari ≥ 40 years old | 0.07 (0.12) a | 0.00 (0.00) b |
| IE Women Otamari < 40 years old | 0.09 (0.16) a | 0.00 (0.00) b |
| IE Women Waama | 0.24 (0.21) a | 0.09 (0.16) b |
| IE Women Waama ≥ 40 years old | 0.24 (0.21) a | 0.00 (0.00) b |
| IE Women Waama < 40 years old | 0.24 (0.21) a | 0.09 (0.16) b |
| Total IE for Men | 0.79 (0.21) a | 0.85 (0.19) a |
| IE Men Berba | 0.19 (0.33) a | 0.30 (0.52) b |
| IE Men Berba ≥ 40 years old | 0.19 (0.33) a | 0.30 (0.52) b |
| IE Men Berba < 40 years old | 0.12 (0.21) a | 0.00 (0.00) b |
| IE Men Otamari | 0.09 (0.16) a | 0.12 (0.21) b |
| IE Men Otamari ≥ 40 years old | 0.09 (0.16) a | 0.12 (0.21) b |
| IE Men Otamari < 40 years old | 0.09 (0.16) a | 0.03 (0.05) b |
| IE Men Waama | 0.59 (0.53) a | 0.48 (0.43) ab |
| IE Men Waama ≥ 40 years old | 0.57 (0.51) a | 0.39 (0.34) ab |
| IE Men Waama < 40 years old | 0.31 (0.27) a | 0.15 (0.26) b |

In a single column, for each index, the values followed by the same letter are not significantly different (Kruskal-Wallis test)
