# Supplementary material for: Ethnic differences in use values and use patterns of Parkia biglobosa in Northern Benin
Source: J Ethnobiol Ethnomed. 2011 Dec 7;7:42. doi: 10.1186/1746-4269-7-42 (PMC3251525; doi:10.1186/1746-4269-7-42)
Supplement: Additional file 4 — Quantitative measurements of knowledge about P. biglobosa in Donga Department. The additional file 1; 2; 3 and 4 give more detail on how the data were analyzed by ethnic group, gender group and age group. [file 1746-4269-7-42-S4.PDF]

Additional file 4 Quantitative measurements of knowledge about *P. biglobosa* in Donga Department

	Farmers	Traditional Healers
Total number of interviewees	488	41
Number of uses cited	76	47
Interviewee diversity value (ID)	Mean (Standard deviation)	Mean (Standard deviation)
Total ID	0.61 (0.08) a	0.42 (0.35) a
Total ID for Women	0.00 (0.00) b	0.01 (0.02) a
ID Women Nago	0.00 (0.00) b	0.01 (0.02) a
ID Women Nago ≥ 40 years old	0.00 (0.00) b	0.01 (0.02) a
ID Women Nago < 40 years old	0.00 (0.00) b	0.00 (0.00) a
Total ID for Men	0.61 (0.08) a	0.42 (0.35) a
ID Men Anii	0.13 (0.23) b	0.12 (0.21) a
ID Men Anii ≥ 40 years old	0.12 (0.21) b	0.11 (0.18) a
ID Men Anii < 40 years old	0.05 (0.09) b	0.03 (0.05) a
ID Men Nago	0.11 (0.14) b	0.22 (0.38) a
ID Men Nago ≥ 40 years old	0.11 (0.14) b	0.19 (0.33) a
ID Men Nago < 40 years old	0.04 (0.07) b	0.06 (0.11) a
ID Men Lokpa	0.36 (0.21) a	0.06 (0.10) a
ID Men Lokpa ≥ 40 years old	0.29 (0.19) b	0.03 (0.06) a
ID Men Lokpa < 40 years old	0.21 (0.11) b	0.03 (0.05) a
ID Men Yom	0.16 (0.20) b	0.09 (0.16) a
ID Men Yom ≥ 40 years old	0.14 (0.18) b	0.08 (0.13) a
ID Men Yom < 40 years old	0.08 (0.08) b	0.04 (0.07) a
ID Men Foodo	0.11 (0.15) b	0.00 (0.00) a
ID Men Foodo ≥ 40 years old	0.09 (0.12) b	0.00 (0.00) a
ID Men Foodo < 40 years old	0.04 (0.07) b	0.00 (0.00) a
Interviewee equitability value (IE)	Mean (Standard deviation)	Mean (Standard deviation)
Total IE	0.87 (0.11) a	0.51 (0.43) a
Total IE for Women	0.00 (0.00) b	0.02 (0.03) a
IE Women Nago	0.00 (0.00) b	0.02 (0.03) a
IE Women Nago ≥ 40 years old	0.00 (0.00) b	0.02 (0.03) a
IE Women Nago < 40 years old	0.00 (0.00) b	0.00 (0.00) a
Total IE for Men	0.87 (0.11) a	0.51 (0.43) a
IE Men Anii	0.19 (0.33) b	0.14 (0.25) a
IE Men Anii ≥ 40 years old	0.18 (0.30) b	0.13 (0.22) a
IE Men Anii < 40 years old	0.07 (0.13) b	0.03 (0.06) a
IE Men Nago	0.16 (0.20) b	0.26 (0.46) a
IE Men Nago ≥ 40 years old	0.16 (0.21) b	0.23 (0.40) a
IE Men Nago < 40 years old	0.06 (0.10) b	0.08 (0.13) a
IE Men Lokpa	0.52 (0.30) a	0.07 (0.12) a
IE Men Lokpa ≥ 40 years old	0.42 (0.28) b	0.04 (0.07) a
IE Men Lokpa < 40 years old	0.30 (0.16) b	0.03 (0.06) a
IE Men Yom	0.23 (0.28) b	0.11 (0.19) a
IE Men Yom ≥ 40 years old	0.21 (0.25) b	0.09 (0.16) a
IE Men Yom < 40 years old	0.11 (0.11) b	0.05 (0.09) a
IE Men Foodo	0.16 (0.21) b	0.00 (0.00) a
IE Men Foodo ≥ 40 years old	0.13 (0.17) b	0.00 (0.00) a
IE Men Foodo < 40 years old	0.06 (0.10) b	0.00 (0.00) a

In a single column, for each index, the values followed by the same letter are not significantly different (Kruskal-Wallis test)
